# Nature of bacitracin resistance in *Staphylococcus aureus*

**DOI:** 10.1128/aac.00875-25

**Published:** 2025-11-04

**Authors:** Merianne Mohamad, Mollie M. Hurst, Elham Elkrewi, Christopher P. Randall, Alex J. O’Neill

**Affiliations:** 1 School of Molecular and Cellular Biology, Faculty of Biological Sciences, University of Leeds 4468, Leeds, United Kingdom; The Peter Doherty Institute for Infection and Immunity, Melbourne, Victoria, Australia

**Keywords:** bacitracin, peptide antibiotic, topical antibacterial, triple antibiotic ointment, resistance determinants, silencing of antibiotic resistance by mutation (SARM)

## Abstract

Bacitracin is employed for topical treatment of staphylococcal infection, though information is lacking regarding the nature of resistance to this antibiotic in the staphylococci. Here we examined bacitracin resistance in a large collection (*n* = 1,470) of multidrug-resistant isolates of *Staphylococcus aureus*. Susceptibility testing of the entire collection revealed a broad range of bacitracin minimum inhibitory concentrations (MICs) (from 32 to >4,096 µg/mL) and allowed us to define a tentative epidemiological cut-off value (TECOFF) (apparent upper end of the wild-type distribution in terms of bacitracin susceptibility) of 256 µg/mL. On the basis of this TECOFF, 101 strains (6.8% of the total) for which bacitracin had an MIC of ≥512 µg/mL were considered resistant. Bacitracin resistance was found across multiple sequence types (STs), though over half the resistant strains belonged to the ST8:USA300 lineage. In nearly all bacitracin-resistant strains (99 of 101), whole genome sequence analysis identified operons similar to the *bcrABD* operon that confers bacitracin resistance in *Enterococcus faecalis*; 3 strains carried an operon [*bcrAB*(ISL3)*D*] closely related to the latter (~90% identity in the encoded resistance proteins), while 96 strains harbored a more distantly related operon (<50% identity in the encoded proteins) that we distinguished with the designation *bcrEFH*. Molecular cloning experiments confirmed the ability of both *bcr* operons to confer bacitracin resistance in *S. aureus*. Both *bcrAB*(ISL3)*D* and *bcrEFH* reside on IS*6*-flanked pseudo-compound transposons on multidrug resistance plasmids, highlighting their potential to spread and become more widely disseminated among staphylococci.

## INTRODUCTION

Bacitracin is an antibiotic complex comprising closely related cyclic polypeptides produced by strains of the genus *Bacillus* (1). The bactericidal action of this agent involves inhibition of cell-wall biosynthesis; bacitracin binds to undecaprenyl pyrophosphate (UPP), the lipid carrier required for the translocation of peptidoglycan precursors across the cytoplasmic membrane, and prevents the dephosphorylation event necessary for recycling of the carrier (2, 3). Since its discovery in the mid-1940s, bacitracin has been employed therapeutically against bacterial infection in humans in several ways, but its primary application has been in the form of a topical agent for the prevention and treatment of superficial infection (4). Use of bacitracin in this context harnesses the narrow-spectrum activity of the antibiotic against gram-positive bacteria to target the pathogens most commonly responsible for such infections (predominantly staphylococci, such as *Staphylococcus aureus*, and to a lesser extent, *Streptococcus pyogenes*) (4).

Despite a long history of deployment for preventing/treating staphylococcal infection, little information exists regarding acquired resistance to bacitracin in this genus. Only a handful of small surveys have reported the presence of reduced bacitracin susceptibility in *S. aureus* (5–7), and such studies are hampered in their interpretation by the lack of a precise definition for bacitracin resistance in this species (4). There is also a lack of information about the genetic basis for bacitracin resistance in staphylococci. More than one report has detected the presence of the *bcrABD* operon in *S. aureus* (8, 9), a determinant that has been shown to confer resistance to bacitracin in *Enterococcus faecalis* (10) and *Clostridium perfringens* (11). However, the role played by this operon in determining bacitracin resistance among clinical isolates of *S. aureus* remains to be established.

Here, we explored the nature of bacitracin resistance in *S. aureus* in a large collection (*n* = 1,470) of multidrug-resistant strains.

## MATERIALS AND METHODS

### Bacterial strains and bacitracin susceptibility testing

The collection of *S. aureus* isolates used here was assembled and subjected to whole genome sequencing (WGS) in a previous study (BioProject accession number PRJEB13165) (12). Multilocus sequence typing of select strains was performed (https://cge.food.dtu.dk/services/MLST/) using genome sequence data (13). Minimum inhibitory concentration (MIC) values for bacitracin (Sigma-Aldrich, product code 11702) were determined by agar dilution according to Clinical and Laboratory Standards Institute guidelines (14) using cation-adjusted Mueller-Hinton agar (MHA, Oxoid), with *S. aureus* SH1000 (15, 16) employed as a bacitracin-sensitive control strain. ECOFFinder (17, 18) was used to define a tentative epidemiological cut-off value (TECOFF) for bacitracin susceptibility in *S. aureus*.

### Investigating the genetic basis of bacitracin resistance

The encoded protein products of the *bcrABD* operon from *E. faecalis* AR01/DGVS (accession number AY496968) were used to interrogate whole genome sequences of all *S. aureus* strains using TBLASTN. For molecular cloning of *bcrAB*(ISL3)*D*, the operon was PCR-amplified from strain MOS184 using oligonucleotide primers 5′-GGCTTCGAAATGGATTATATCATTGA and 5′-GCGGTTCGAAAATCAAAGGACTATTAGG (engineered restriction sites underlined) and digested with BstBI (NEB) to enable ligation into similarly digested shuttle plasmid, pSK5487M (19, 20). Cloning of the *bcrEFH* determinant was achieved using this same plasmid, though the presence of internal BstBI sites in this operon instead necessitated a blunt-end cloning approach; a PCR amplicon was generated from strain NRS384 using 5*′* phosphorylated oligonucleotide primers 5′-GGCGGGTTGAATAATATAATTGAA and 5′-GCTACAGTGATAACATCCTTGT and ligated into BstBI-cut and blunted pSK5487M. Ligation mixtures were used for electrotransformation (21) of *S. aureus* RN4220 (22), and the resulting recombinant constructs confirmed by DNA sequencing.

To select bacitracin-resistant revertants from strains carrying a genetically inactivated version of the *bcrEFH* operon, saturated cultures were plated onto MHA containing bacitracin at 4× the corresponding MIC and incubated for 48 h at 37°C. Total cell counts were determined in parallel on antibiotic-free agar, enabling calculation of reversion frequencies (no. of revertants /total no. of bacteria). Apparent bacitracin-resistant revertants were confirmed by susceptibility testing and PCR amplification/DNA sequencing of *bcrE* using primers 5′-CAAAGCAATATGGAAGTCAGATAGCAG and 5′- CTATCACTCTCATCTGGAAAACATCAATATTG.

DNA sequencing of plasmids harboring bacitracin-resistance determinants was performed by CeGat (Tübingen, Germany). Plasmid preparations from strains MOS184, NRS19, and NRS384 were first used to transform *S. aureus* RN4220 to bacitracin resistance, and plasmid DNA from transformants then recovered for sequencing; this approach sought to ensure that samples for DNA sequencing contained only the plasmid of interest. Sequence reads were assembled using SPAdes (23).

## RESULTS

### Defining bacitracin resistance and gauging prevalence/distribution across sequence types

**Fig 1:**
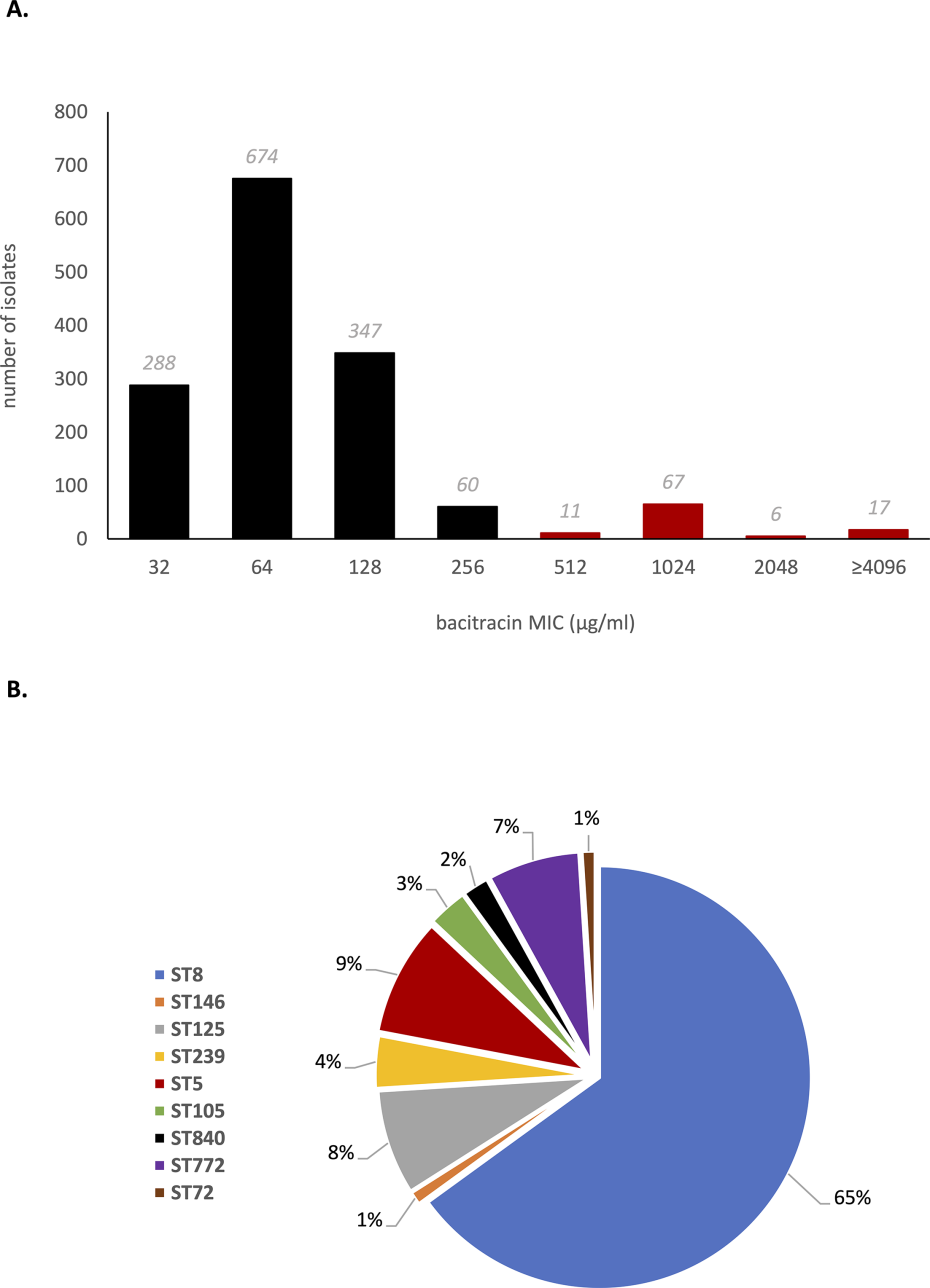
Bacitracin susceptibility of 1,470 clinical isolates of *Staphylococcus aureus* (**A**) and distribution of sequence type among resistant isolates (**B**). In panel **A**, isolates considered bacitracin resistant (on the basis of a TECOFF of 256 µg/mL) are shown in red. While all MIC values are reported here exactly as measured, it should be noted that at higher concentrations, the apparent activity of bacitracin may become limited by the availability of the divalent cations in the growth medium that are essential for antibacterial action (24); hence, we caution against overinterpretation of precise MIC values/differences at the upper end of the range.

A previously assembled collection of 1,470 multidrug-resistant *S. aureus* isolates (12) was subjected to bacitracin susceptibility testing. Bacitracin MICs spanned a broad range, from 32 to >4,096µg/mL (Fig. 1A). No clinical breakpoints exist to discriminate bacitracin-susceptible and bacitracin-resistant *S. aureus*, and we therefore took the approach of determining a TECOFF to define the upper end of the wild-type (antibiotic susceptible) MIC distribution. Analysis of our MIC data using ECOFFinder (17, 18) returned a bacitracin TECOFF of 256µg/mL, indicating that strains for which bacitracin has an MIC of ≥512 µg/mL should be considered resistant. On that basis, 101 of the 1,470 strains (~6.8%) tested in this study were deemed bacitracin resistant (Fig. 1A).

A previous study reported reduced bacitracin susceptibility to be a common property of the USA300 lineage of methicillin-resistant *Staphylococcus aureus* (MRSA) (6). We therefore examined whether any of our bacitracin-resistant strains belonged to this lineage. Of 101 bacitracin-resistant strains, 66 were found to be sequence type (ST) 8, with basic local alignment search tool (BLAST) analysis detecting the presence of USA300-hallmark genes (*lukSF-PV* and ACME) in most of these (56 out of 66); thus, over half of the bacitracin-resistant strains in this collection are USA300. Nevertheless, bacitracin resistance is not restricted to USA300, with the remaining resistant strains belonging to a variety of STs (ST5 [*n* = 9], ST125 [*n* = 8], ST772 [*n* = 7], ST239 [*n* = 4], ST105 [*n* = 3], ST840 [*n* = 2], ST72 [*n* = 1], and ST146 [*n* = 1]) (Fig. 1B).

### The *bcr* genes are the predominant bacitracin-resistance determinants in *S. aureus*

**Fig 2:**
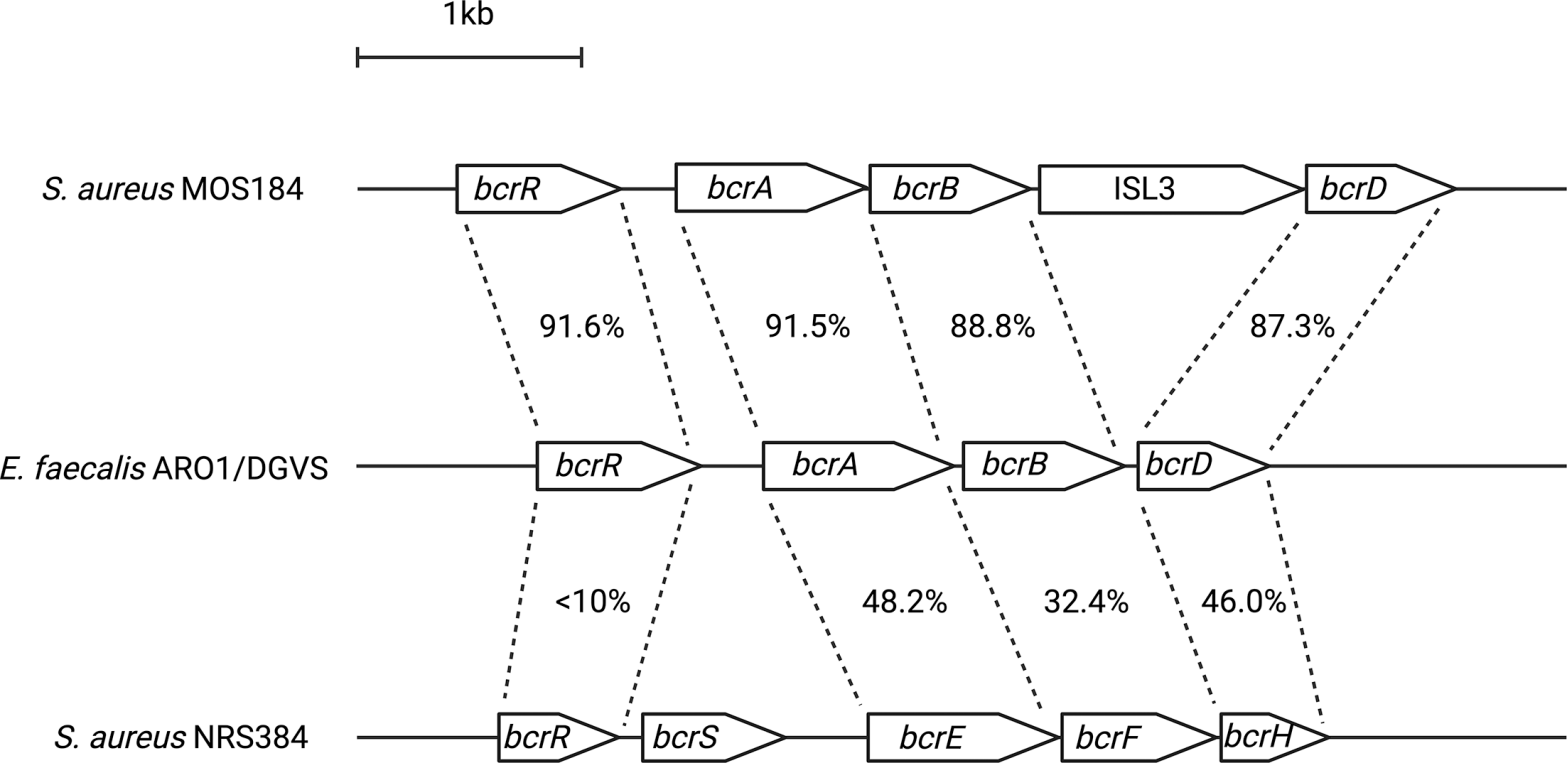
Comparison of the architecture and encoded protein identity between the *bcrABD* operon from *E. faecalis* ARO1/DCVS (accession number AY496968) and the *bcrAB*(ISL3)*D* and *bcrEFH* operons identified in *S. aureus*.

As indicated above, the *bcrABD* operon that confers bacitracin resistance in other gram-positive bacteria has been detected in *S. aureus*, including in strains exhibiting reduced bacitracin susceptibility (8, 9). While it has not been experimentally confirmed that this operon is responsible for bacitracin resistance in such strains, it seemed likely that this is indeed the case. On that basis, we interrogated the genome sequences of all 1,470 strains for the presence of *bcr* operons by TBLASTN using proteins encoded by the *bcrABD* operon of bacitracin-resistant *E. faecalis* AR01/DGVS (accession number AY496968) (10). This analysis detected the same *bcr* operon in three bacitracin-resistant *S. aureus* strains (MOS1, MOS62, and MOS184). While this operon is closely related to *E. faecalis bcrABD*, with the encoded proteins exhibiting ~90% identity to their enterococcal counterparts (Fig. 2), it carries an ISL3 insertion sequence between *bcrB* and *bcrD*; consequently, we refer to it as *bcrAB*(ISL3)*D*. As seen for the *bcrABD* operon from *E. faecalis*, *bcrAB*(ISL3)*D* lies immediately downstream of a regulatory gene (*bcrR*) (Fig. 2). In a further 96 bacitracin-resistant *S. aureus* strains, we detected a distantly related *bcr* operon whose encoded proteins exhibit a much lower degree of identity (<50%) with *E. faecalis* BcrABD (Fig. 2). To distinguish this latter operon from the more canonical *bcrAB*(ISL3)*D*, we designated it *bcrEFH*. Immediately upstream of this operon lie two genes (designated *bcrS* and *bcrR*) that appear to encode the histidine kinase and response regulator, respectively, of a two-component regulatory circuit (Fig. 2).

The fact that *bcr* operons were present in 99 of 101 bacitracin-resistant strains but near-universally absent from bacitracin-susceptible strains (see below) suggested that they are responsible for most cases of bacitracin resistance in our collection. To corroborate this idea, we sought to experimentally link *bcrAB*(ISL3)*D* and *bcrEFH* with bacitracin resistance by introducing these operons independently into a bacitracin-susceptible laboratory strain (*S. aureus* RN4220) on pSK5487M, a plasmid that carries the moderate-strength, constitutive P*_qacR_* promoter to drive gene expression. While *S. aureus* RN4220 carrying the empty plasmid backbone showed no change in bacitracin susceptibility (MIC of 64µg/mL), strains carrying either *bcrAB*(ISL3)*D* or *bcrEFH* exhibited bacitracin resistance (MIC of >1,024µg/mL, comparable to that observed against *S. aureus* strains that natively carry these operons).

Thus, bacitracin resistance in clinical strains of *S. aureus* is typically conferred by two distinct types of *bcr* operon, of which *bcrEFH* appears to be the most prevalent. The latter was detected across a broad range of STs (listed above), while *bcrAB*(ISL3)*D* was exclusively found in ST239. The genetic basis for bacitracin resistance could not be established in 2 of the 101 resistant strains; the genome sequences of these organisms did not include *bcrAB*(ISL3)*D*, *bcrEFH*, or a related *bcr* operon, and in neither case could the bacitracin-resistance phenotype be transferred by transduction or transformation into a laboratory strain (*S. aureus* RN4220) for further characterization (data not shown). It therefore appears that additional, non-*bcr-*type bacitracin-resistance determinants exist in *S. aureus*.

### Detection and characterization of “silenced” bacitracin resistance

Silencing of antibiotic resistance by mutation (SARM) describes a recently dissected phenomenon (12) in which bacteria carry a resistance determinant but appear sensitive to the corresponding antibiotic as a consequence of a genetic defect. SARM is potentially of major clinical importance because while such strains appear antibiotic sensitive, reversion of the silencing mutation can readily occur to restore phenotypic resistance (12). We identified three strains (MOS300, MOS177, and NRS736) in our collection that exhibited SARM in respect to bacitracin; all carry the *bcrEFH* operon but were bacitracin susceptible (MIC of ≤128µg/mL). In all three strains, the *bcrE* gene appeared to be inactivated by a frameshift mutation owing to a single adenosine deletion at a poly(A) tract starting at nucleotide position 664. To assess the ability of these strains to undergo reversion to bacitracin resistance, saturated cultures were challenged with bacitracin at 4× MIC on agar. Bacitracin-resistant revertants (MIC of >1,024 µg/mL) were readily recovered from all three strains, with similar reversion frequencies observed in all cases (~2 to 3 × 10^−8^). DNA sequencing of the *bcrE* gene from selected revertants from each strain revealed reinstatement of an adenosine nucleotide in the poly(A) tract from which it had originally been lost (i.e., restoration of bacitracin resistance by direct reversion).

### *bcr* operons are associated with mobile genetic elements in *S. aureus*

DNA sequence analysis of the immediate genetic environment of *bcrAB*(ISL3)*D* and *bcrEFH* operons in representative strains suggested a plasmid location for these resistance determinants. To confirm this, plasmid preparations from strains MOS184 (*bcrAB*(ISL3)*D^+^*) and NRS384 (*bcrEFH^+^*) were independently used for electroporation of *S. aureus* RN4220, yielding bacitracin-resistant transformants (MIC >512 µg/mL) in both cases. Analysis of the transformants revealed that both carried a plasmid of c. 30 kb, which corresponds in size to plasmids present in MOS184 and NRS384 (data not shown). Since we were unable to compile the complete circular DNA sequence of these plasmids from the existing WGS data, both bacitracin-resistance plasmids were purified from their corresponding transformants and subjected to whole plasmid sequencing.

**Fig 3:**
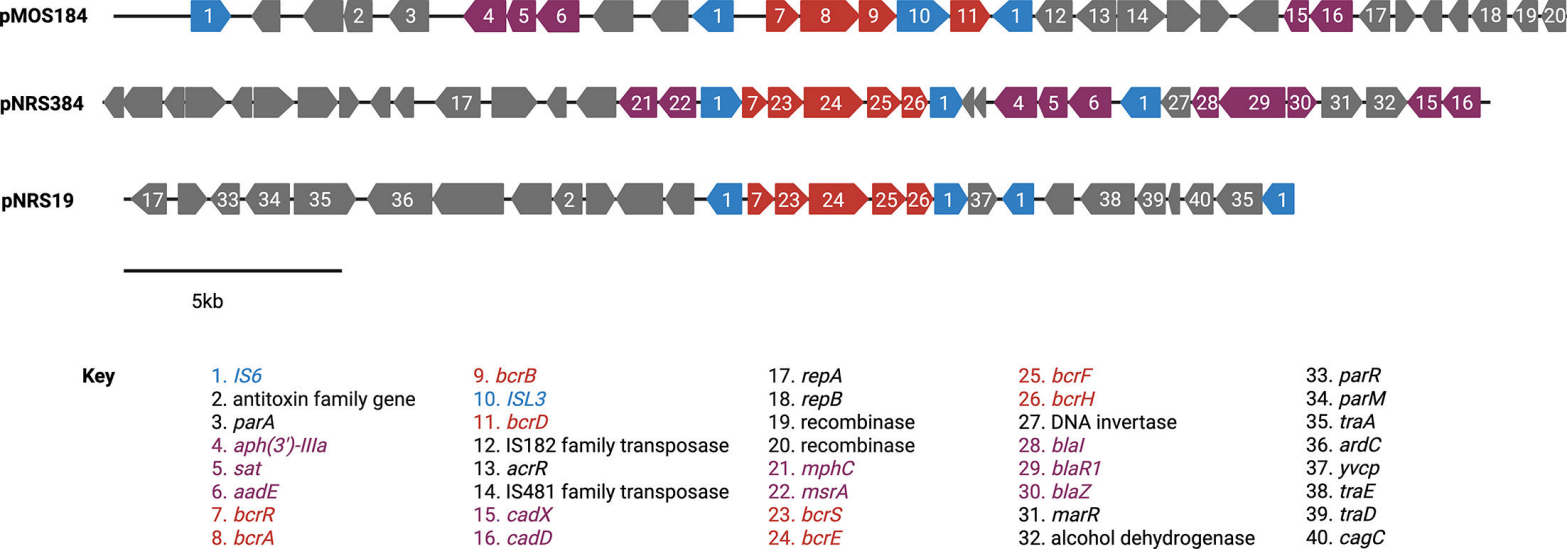
Nature of plasmids carrying *bcrAB*(ISL3)*D* (pMOS184) and *bcrEFH* (pNRS19 and pNRS384). The *bcrAB*(ISL3)*D* and *bcrEFH* operons are indicated in red, other antimicrobial resistance genes in purple, and insertion sequences (IS*6* and ISL3) in blue. Other genes with known or likely functions are labeled accordingly.

The plasmid from MOS184 (designated pMOS184 [GenBank accession number PV454381]) is ~32.8 kb in size and, in addition to *bcrAB*(ISL3)*D*, harbors an aminoglycoside-streptothricin resistance gene cluster (*aphA*, *sat*, and *aadE*) and a cadmium resistance operon (*cadDX*) (Fig. 3). Plasmid pMOS184 lacks *tra* genes, indicating it is non-conjugative. The *bcrAB*(ISL3)*D* operon is flanked by IS*6* insertion sequences (Fig. 3), yielding a pseudo-compound transposon; this implies that this bacitracin-resistance determinant is mobile beyond its association with a plasmid. BLAST analysis using pMOS184 against WGS data from the other two strains that carry *bcrAB*(ISL3)*D* (MOS1 and MOS62) established that the same—or a very closely related —plasmid is present in all three strains (data not shown).

The plasmid from NRS384 (designated pNRS384 [GenBank accession number PV454382]) that carries *bcrEFH* has features in common with pMOS184; it is a similar size (~32.0 kb), harbors aminoglycoside-streptothricin and cadmium resistance determinants, is non-conjugative (*tra*^−^), and the bacitracin-resistance determinant itself resides on an IS*6*-flanked pseudo-compound transposon (Fig. 3). Nevertheless, they are not closely related plasmids; pNRS384 additionally confers resistance to macrolides (via *msrA* and *mphC*) and beta-lactams (via the *bla* operon), and sequence alignment of pNRS384 with pMOS184 revealed <40% sequence identity across their entire lengths (data not shown).

To determine if pNRS384 or closely related plasmids are carried by other *bcrEFH^+^* strains in our collection, we undertook BLAST analysis of the linearized plasmid against WGS data of representatives of all distinct sequence types in the collection that harbor *bcrEFH*: ST8 (MOS309 and MOS205); ST125 (MOS308, MOS16, and MOS317); ST5 (NRS19, NRS656, and NRS767); ST772 (DUB3 and DUB29); ST72 (NRS386); ST105 (NRS738, NRS837, and NRS838); and ST840 (NRS675 and NRS714). With one exception—strain NRS19—all strains analyzed possessed >70% of the pNRS384 plasmid sequence, including the regions encompassing the *bcrEFH* operon and the other resistance genes (data not shown). Thus, almost all of the *bcrEFH*^+^ strains carry pNRS384 or a related plasmid.

WGS data from strain NRS19 returned <40% sequence identity alignment to pNRS384 and did not therefore carry a pNRS384-like plasmid. As above, transformation experiments were conducted to confirm that the *bcrEFH* operon resides on a plasmid in this strain, and DNA sequencing was then used to characterize the plasmid (designated pNRS19 [GenBank accession number PV454383]). Plasmid pNRS19 is a ~26.5 Kb conjugative (*tra*^+^) plasmid, on which *bcrEFH* is the sole antimicrobial resistance determinant (Fig. 3). The DNA sequence of the *bcrEFH* operon and its upstream regulatory genes in pNRS19 exhibits >99% identity to that present on pNRS384 and is likewise flanked by IS*6* elements.

## DISCUSSION

On the basis of the bacitracin TECOFF defined here (256 µg/mL), this study identified acquired resistance to bacitracin in 6.8% of a large collection of *S. aureus* strains. Since the collection in question is composed entirely of multidrug-resistant strains (defined as resistant to two or more clinically deployed antistaphylococcal drug classes)—with most (∼86%) being MRSA—this prevalence estimate likely represents a worst-case scenario and is no substitute for more formal surveillance. Nevertheless, it is clear that bacitracin resistance is not rare in *S. aureus* and exists across a range of sequence types.

Corroborating the ability of the proposed TECOFF to appropriately distinguish *S. aureus* strains that have acquired resistance to bacitracin, a bacitracin-resistance determinant was detected in almost all (99 of 101) strains in this study for which bacitracin had an MIC of ≥512 µg/mL. Bacitracin resistance was near universally attributable to carriage of *bcr*-type operons, which were found to exist in two forms: the *bcrAB*(ISL3)*D* operon that is closely related to the *bcrABD* determinant responsible for bacitracin resistance in enterococci, and the more distantly related *bcrEFH* operon. Of the two, the latter appears far more prevalent, present in 95% of the bacitracin-resistant *S. aureus* strains identified in this study. Both of these staphylococcal *bcr* operons are associated with mobile genetic elements that likely facilitate their spread; in all examples characterized in this study, these operons reside on transposon-like elements that are themselves carried by plasmids, and the specific case of pNRS19 reveals that *bcrEFH* also exists on plasmids that are conjugative (i.e., self-transmissible).

The plasmids harboring *bcr* operons typically also carry other antimicrobial resistance genes; we consider it potentially significant that aminoglycoside resistance determinants are a near-universal feature of these plasmids. In countries that include the United States and Canada, bacitracin is commonly deployed as part of an over-the-counter preparation known as triple antibiotic ointment (TAO) that also contains polymyxin B and an aminoglycoside (neomycin) (25). Polymyxin B has limited effect against gram-positive bacteria such as *S. aureus*, and the antistaphylococcal activity of TAO predominantly results from the concerted action of bacitracin and neomycin. The observation that bacitracin-resistance determinants in *S. aureus* almost invariably reside on plasmids that also confer neomycin resistance (neomycin MICs of ≥256 µg/mL for pMOS184 or pNRS384 in *S. aureus* RN4220) could suggest that TAO use is selecting co-resistance to both drugs. To what extent the presence of a bacitracin-neomycin resistance plasmid will serve to compromise the therapeutic utility of TAO against *S. aureus* remains to be established.

The mechanism by which the encoded products of the *bcr* operons mediate bacitracin resistance in *S. aureus* will require further investigation. Bcr-type systems were first identified as a mechanism of self-protection in a bacitracin producer organism (*Bacillus licheniformis*), where it was noted that the BcrA and BcrB proteins, respectively, resemble the ATP-binding domain and membrane spanning portions of an ABC transporter, prompting the idea that Bcr-type resistance involves active efflux of bacitracin (26). However, given that bacitracin acts at the extracellular surface of the cytoplasmic membrane, it is not obvious how a “classical” efflux system might operate in this context or how it would serve to hinder bacitracin action. More likely, Bcr-type systems work by target protection (27), a mechanism that has been proposed for the unrelated BceAB “transporter” that provides intrinsic protection against bacitracin and other peptide antibiotics in *Bacillus subtilis*. BceAB appears to recognize antibiotic-target complexes at the cell surface, with consequent ATP hydrolysis providing the necessary energy to catalyze dissociation of the antibiotic and thereby free the target from inhibition (28).

The role of the third gene of *bcr* operons (*bcrD* and *bcrH*) also remains to be clarified. The encoded products of these genes appear to be undecaprenol kinases that could theoretically help counter the inhibitory action of bacitracin by generating undecaprenyl monophosphate to enable continued cell-wall synthesis when UPP recycling is blocked by the antibiotic (10). In practice, no significant reduction was observed in the level of bacitracin resistance conferred by the *bcrABD* operon in *E. faecalis* when the *bcrD* gene was removed, and expression of *bcrD* alone in the absence of *bcrAB* did not confer any reduction in bacitracin susceptibility (10); we observed a comparable situation in *S. aureus* (data not shown). While therefore not enhancing the level of bacitracin resistance conferred by *bcr* operons, we speculate that *bcrD/H* might instead be acting to improve the efficiency with which resistance is achieved, mitigating to some extent the energetic burden incurred by the cell if a BcrAB-type system were alone responsible for providing protection from bacitracin and making heavy use of ATP in the process.
